# Factors Associated with Pediatric Emergency Room Utilization in an Urban Community Hospital in Santiago, Dominican Republic

**DOI:** 10.5334/aogh.2327

**Published:** 2019-06-06

**Authors:** Allison Lockwood, Aparna Dandekar, Margaret Arias, Massiel Ovalles, Suzanne Bentley

**Affiliations:** 1 Icahn School of Medicine at Mount Sinai, New York, US; 2 Department of Family Medicine, Mount Sinai Hospital, New York, US; 3 Department of Health System Design and Global Health, Icahn School of Medicine at Mount Sinai, New York, US; 4 Department of Family Medicine, Hospital Especializado Juan XIII, Santiago, DR; 5 Department of Emergency Medicine, Elmhurst Hospital, Icahn School of Medicine at Mount Sinai, New York, US

## Abstract

**Background:**

In low resource settings, maximizing effective use of emergency room (ER) services is imperative. This problem is anecdotally observed in the public hospital setting in Santiago, Dominican Republic (DR). There are no studies presently published examining ER use in this pediatric population or reasons caregivers choose to utilize the pediatric ER. Financial and systemic limitations have been previously cited as important contributors to the high pediatric mortality rate in the DR.

**Methods:**

In this cross-sectional, descriptive study, a survey was administered to 117 caregivers (e.g. mother, father) of children in the ER at Hospital Especializado Juan XXIII over an eight-week period. Survey questions included perceived urgency of illness, education level, monthly income, and frequency of ER visits in the last six months. We defined frequent ER visits as greater than four visits within the last six months, low income as below 10,000 pesos/month, and low education as having no high school education. Logistic regression was used to assess significant associations between variables.

**Results:**

Caregivers in the pediatric ER were predominantly female (94%) with a mean age of 30 ± 11 years. 72% of respondents reported the child’s problem as “extremely urgent,” while 82% of the children were triaged as non-urgent. Children of caregivers with any high school education had 69% lower odds of having 4 or more ED visits in the last 6 months (OR, 0.31; 95% CI, 0.13–0.75; p = 0.009), compared to children of caregivers with no high school education, after adjusting for the income category of the caregiver.

**Conclusions:**

Perceived urgency of pediatric medical problems may contribute to increased use of the ER for non-urgent medical problems. Low education level is associated with increased pediatric ER use over a six-month period. Assessing utilization of pediatric ERs in the public health care system in Santiago could provide a framework for the design of targeted educational and systemic changes, supporting the ultimate goal of providing the best possible care for pediatric patients in low-resource settings.

## Introduction

Pediatric emergency department (ED) use for non-urgent medical complaints is a well-recognized problem in the United States [1,2,3]. Hospital and ED overcrowding has been described as an international crisis that may affect the quality and access of health care [4,5]. This problem is anecdotally observed in the public hospital setting in Santiago, Dominican Republic (DR). There are no studies presently published examining the reasons why local caregivers may choose to utilize the pediatric ED for non-urgent medical problems.

Socioeconomic status and systemic healthcare limitations have been cited as important contributors to the high pediatric mortality rate in the DR [6,7]. Maternal education, insurance status, age of the child, duration of illness, and nutritional status have all been identified as predictors of children’s health services utilization in the DR [8,9]. United States-based research has shown a decrease in pediatric ED usage as a result of parental education interventions [10,11]. It is important to understand how and why caregivers access emergency services in order to allocate resources appropriately.

The primary objective of this preliminary, exploratory study at a small urban community public hospital in Santiago, DR is to determine if factors including level of education of the caregiver, monthly income, and health insurance status are associated with use of emergency services at a community hospital in Santiago, DR. Assessing why caregivers utilize pediatric emergency services could help inform the design of future interventions aimed at empowering caregivers to make emergency care seeking decisions for their children. A secondary objective of this study is to investigate reasons why caregivers chose to visit the ED as opposed to primary care clinics located in neighborhoods surrounding the hospital.

## Methods

### Sample

In this cross-sectional, descriptive study, a survey was administered to caregivers (e.g. mother, grandparent) of children in the ED at Hospital Especializado Juan XXIII over an eight-week period from June–July 2015. Eligible subjects were 18 years of age or older who had brought a child to the ED for evaluation. Caregivers with children who were triaged with an illness considered “extremely urgent” were not given the survey and were excluded from the sample. A convenience sample of 117 caregivers was recruited.

### Setting

Hospital Especializado Juan XXIII is a community hospital in Santiago, the second-largest city in the DR. The hospital serves a catchment area of approximately 300,000 people and houses family medicine and community health residency programs. The ED at Hospital Especializado Juan XXIII is staffed predominantly by family medicine residents. Cases in the ED are seen at the behest of the resident staffing the ED that day. Complex cases beyond the capabilities of the ED, such as those requiring advanced airways, are transferred to tertiary care hospitals by taxi or private vehicle. ED services are available 24 hours/day, 7 days/week and free of charge.

The Unidades de Atención Primaria (UNAPs) are small primary care clinics located in the neighborhoods surrounding the hospital. They are open during specific hours each day and require patients to sign up when the clinic opens, then wait for their appointment. UNAP services are free of charge. The UNAPs are also staffed by family medicine residents from Hospital Especializado Juan XXIII. Services available at UNAPs include visits for urgent concerns, primary care, prenatal care, well-baby care, and basic gynecological care for all ages.

### Survey Instrument

Several previously validated questionnaires were consulted [12,13]. The survey instrument was collaboratively developed with Dominican physicians and translated into Spanish at a 6^th^ grade reading level with desired scope and specificity to the DR. The survey addressed 3 categories of questions:

1. Past and present ED use–current chief concern, length of illness, treatments given at home (medications or home remedies), frequency of hospital visits in the past 6 months, perceived urgency of illness, time between home and closest hospital, and time between home and UNAP.
2. Past education about common childhood illnesses–if a medical professional had ever spoken with the subject about what to do if their child had a fever, vomiting, diarrhea, or trouble breathing. Questions were phrased as follows:
  - Has a doctor, nurse, or community health worker ever talked to you about what to do when a child has a fever?
    - Yes
      - What have you been told to do? Fill in the blank______
    - No
3. Sociodemographic characteristics of caregivers–age, gender, nationality, relation to the child, highest level of education attained, marital status, health insurance status, monthly income, and neighborhood of residence.

The survey was written in English and translated into Spanish and Haitian Creole using translators from the DR (Spanish) and Haiti (Haitian Creole). The survey was finalized by Spanish and Haitian Creole-speaking doctors in the DR. The survey was piloted and revised further after administration to 61 subjects to exclude redundant and superfluous questions, in addition to removal of questions that could not be answered appropriately given the ED setting. For example, a question asking about the quality of the service provided at the hospital was removed due to probable discomfort experienced by patients as the hospital staff is continuously present.

### Data Collection

The survey was administered in Spanish by a non-native, fluent medical student researcher with assistance from Dominican residents and in Haitian Creole by Haitian residents and medical student researchers. Surveys were administered during daytime hours. Researchers explained the objective of the study and asked caregivers if they would be willing to participate. Potential subjects gave verbal consent to participate in the survey. Participants were asked if they would like to read the survey themselves or if they would prefer to have it read to them. All but one out of the 117 participants asked to have the survey read to them. The surveys were delivered in either Spanish or Haitian Creole, as preferred by the caregiver. Surveys were anonymous and each participant was assigned a study ID number. Each survey administration required approximately 10–15 minutes to complete. No medical advice or treatment was given by the researchers during the course of the survey and survey participation did not affect or alter care provided in the ED. The study was approved by the Icahn School of Medicine at Mount Sinai Institutional Review Board and by the medical director of Hospital Especializado de Salud Juan XXIII.

### Definitions and Data Analysis

Based on prior studies, low education was defined as having no high school education and low income as earning less than 10,000 pesos per month (approximately equal to 217 USD). A high number of ED visits was defined as four or more visits within the last six months. Pediatric patients were triaged by family medicine residents at the ED based on a regularly used tri-level triage protocol. Triage levels were categorized as Extremely Urgent, Somewhat Urgent, and Not Urgent based on the resident’s assessment of the patient and corresponding to a previous implemented, color-based triage system in the ED (red = Extremely Urgent, yellow = Somewhat Urgent, green = Not Urgent). The hospital ED triage system was previously developed and implemented based on the World Health Organization’s Emergency Triage Assessment and Treatment protocol and the South African Triage Scale protocol [14,15].

Chi square and logistic regression modeling was used in SPSS to analyze association of factors with frequent ED use.

## Results

### Characteristics of Sample

Table 1 shows the sociodemographic characteristics of the sample. A total of 117 caregivers in the pediatric ED completed the survey. Most caregivers were female (110 of 117; 94%) with a median age of 28 (IQR, 24–35) years. Twenty-seven percent of caregivers reported making 10,000 pesos (217 USD) or more per month, while 47% reported making less than 10,000 pesos per month. Twenty-seven percent were unsure of their family’s monthly income. More than half the sample reported having less than a complete high school education.

**Table 1:** Sample Characteristics: Sociodemographics.


Median Age of Caregiver (years)	28
Gender of Caregiver (Female)	94.0%
Mean Age of Child (years)	4.8
Gender of Child (Male)	50.9%
Caregiver’s Relationship to Child (Parent)	79.3
Income	
>10,000 pesos/month	27%
<10,000 pesos/month	47%
Unsure of income	27%
Education	
Illiterate	0.8%
Primary School (Incomplete)	27.4%
Primary School (Complete)	14.2%
High School (Incomplete)	24.8%
High School (Complete)	17.7%
Technical School	0%
University	15.0%
Marital Status	
Single	34.5%
Civil Union	51.3.6%
Married	9.7%
Separated	0.9%
Divorced	2.7%
Widowed	0.9%
Insurance Status	
Insured	47%
Uninsured	53%
Country of Birth	
Dominican Republic	94.0%
Haiti	6.0%


**Table 2:** Characteristics of Presenting Complaint and ED Use.


Symptoms		Child Seen By Another Doctor Prior to ER Visit	
Fever	40.2%	Yes	25.4%
Vomiting	19.7%	No	74.6%
Diarrhea	12.0%	Caregiver Used Medicine or Remedy at Home Prior to ER Visit	
Problems Breathing	12.0%	Yes	57.3%
Sore Throat	15.4%	No	42.7%
Child Needed Injection	5.1%	ER Visits in Past 1 Month	
Cough	5.1%	0–1 visits	61.0%
Open Wound	11.1%	2–3 visits	31.9%
Broken Bone	6.0%	4–5 visits	7.1%
Stomach Pain	6.0%	6–7 visits	0.0%
Headache	6.0%	8–9 visits	0.0%
Not Eating	4.3%	10+ visits	1.8%
Rash	6.8%	ER Visits in Past 6 Month	
Other	16.2%	0–1 visits	30.8%
Average Duration of Illness (Days)	3.7	2–3 visits	44.7%
Child Sees a Doctor Regularly (1–2 visits/year)		4–5 visits	13.6%
Yes	53.8%	6–7 visits	11.7%
No	46.2%	8–9 visits	0.0%
		10+ visits	8.7%


**Table 3:** Urgency of Presenting Complaint.


Perceived Urgency of Illness		Child Triage Level	
Extremely Urgent	65%	Extremely Urgent	Excluded
Somewhat Urgent	12.8%	Somewhat Urgent	14.5%
Not Urgent	12.8%	Not Urgent	68.4%
No Response	8.5%	No Response*	17.1%


* Resident did not assign a triage level, but level of Somewhat Urgent or Not Urgent was utilized as child was not admitted and showed no signs corresponding to Extremely Urgent.

The vast majority of caregivers were born in the DR (94%) with a minority of caregivers born in the neighboring country of Haiti (6%).

### Characteristics of Presenting Complaint and ED Use

The most common presenting complaints reported by caregivers of pediatric patients were fever (40.2%), vomiting (19.7%), sore throat (15.4%), diarrhea (12.0%), and problems breathing (12%). Of caregivers, 53.8% reported that their child had a regular doctor that they saw 1–2 times per year and 25.4% of caregivers reported seeing or calling this doctor prior to their current ED visit; 61% of caregivers reported 0–1 ED visits in the past one month. The majority of caregivers reported two or more ED visits within the past six months.

**Table 4:** Reasons for Visiting ER Instead of UNAP.


ER is faster	7.60%
ER is closer	18.50%
Went to UNAP first	6.50%
ER is better	10.90%
UNAP doesn’t provide the service needed	31.50%
No UNAP in neighborhood	6.50%
Misinformation about UNAPs	10.90%
Other	7.60%


**Table 5:** Distance to Care.


Distance to Nearest Doctor	
1–10 minutes	58.1%
11–20 minutes	21.4%
21–30 minutes	11.1%
41–40 minutes	1.7%
40+ minutes	4.3%
Distance to Nearest UNAP	
1–10 minutes	42.7%
11–20 minutes	17.1%
21–30 minutes	11.1%
41–40 minutes	0.0%
40+ minutes	0.9%
Unsure	15.4%


The presenting complaint reported by caregivers was described as “extremely urgent” in 65% (76 of 117) of cases versus ED residents assignment of triage level “not urgent” in 68.4% (80 of 117) of the children. The perceived urgency level reported by caregivers and triage level assigned by residents matched in 8.5% (10 of 117) of the cases, all of which were “not urgent.”

Caregivers cited the efficiency, quality, and proximity of the ED as reasons for choosing the ED instead of an UNAP. Forty-two percent of caregivers accessed the ED because of reasons categorized as “services were not offered in the UNAPs” or “because they had misinformation about UNAPs”.

In further analysis employing a multivariable model (Table 6), children of caregivers with any high school education had 69% lower odds of having frequent ED use in the last six months (OR, 0.31; 95% CI, 0.13–0.75; p = 0.009), compared to children of caregivers with no high school education, after adjusting for the income category of the caregiver. Insurance status was not found to be a predictor of frequent ED use.

**Table 6:** Factors Associated with lower ED Visit Frequency.

	Descriptive		Univariable		Multivariable	

	N	No. (%) with high ER use	OR (95% CI)	*P*	OR (95% CI)	*P*

Education						
No high school	50	22 (44)	Reference		Reference	
Any high school	63	13 (21)	0.33 (0.14–0.76)	0.009	0.31 (0.13–0.75)	0.009
Income						
<10,000 pesos/month	47	19 (40)	Reference		Reference	
>10,000 pesos/month	28	6 (21)	0.40 (0.14–1.18)	0.10	0.49 (0.16–1.48)	0.20
Unknown	38	10 (26)	0.53 (0.21–1.33)	0.18	0.43 (0.16–1.14)	0.09

## Discussion

This study suggests that the majority of caregivers accessing the ED at Hospital Especializado Juan XXIII are female, are the parent of the child, and have not graduated high school. Caregivers with a complete high school school education were less likely to report frequent ED use for their child than those with less than a high school level of education. Monthly income and health insurance status were not found to be associated with frequent ED use.

A large number of caregivers reported using the ED because services were not offered in the UNAPs or because they had misinformation about UNAPs (e.g. that they must pay for services or need insurance). For example, many caregivers brought their children to the ED for routine vaccinations because it was thought that vaccinations were unavailable in UNAPs. In reality, UNAPs have the ability to provide vaccinations and many of the other services caregivers cited as not being available, such as radiograph and laboratory orders. When this data was presented to the Department of Family Medicine at Hospital Especializado Juan XXIII, several resident physicians noted that community misperceptions regarding services offered at UNAPs may be due to the irregularity of supply chains to UNAPs, Supplies affected often include syringes needed to deliver injections, vaccinations and family planning methods.

Additionally, 10.9% of respondents opined that the ED was better than UNAPs. Of note, UNAPs are staffed by second year family medicine residents, while the ED (during the duration of the study) was primarily staffed by first year family medicine residents under the supervision of senior residents.

This study has several limitations. First, the findings cannot necessarily be considered generalizable as the convenience sample does not represent the overall population of caregivers in pediatric EDs in the DR or elsewhere. Secondly, selection bias occurred as caregivers of patients who were in significant pain or showed any signs of an extremely urgent illness (whether this classification was corroborated with the resident’s triage assessment or not) were largely excluded from the study because per hospital protocol, these patients were taken into an isolated part of the ED for immediate attention. For Spanish-speaking patients, the survey was delivered by a fluent, but non-native Spanish speaker while the surveys in Haitian were delivered by native speakers. While the survey did not address any expressly confidential topics, sensitive issues such as income may have been difficult to discuss. Notably, many caregivers chose “I don’t know” in response to such questions. These findings should be considered both exploratory and preliminary; a larger random sample should be used in further evaluation. Future studies are needed to isolate additional confounders and further explore factors associated with increased ED utilization.

In conclusion, this information could be useful to governing bodies in order to systematize and publicize services available at UNAPs, and remedy any misperceptions by the public. The Hospital Especializado Juan XXIII staff has since used this data in pursuit of improving services at UNAPs. The disparate perceptions of urgency on the part of caregivers versus ED providers, in addition to the increased frequency of ED visits amongst caregivers with a lower level of education, could indicate an opportunity to further educate caregivers to recognize the signs and symptoms of illnesses needing urgent attention and clarify available UNAP primary care services. Addressing the significant public misperceptions about the healthcare services available in the community could provide an opportunity to direct UNAP and ED utilization in the community.

## References

[1] Meggs W, Czaplijski T and Benson N. Trends in emergency department utilization, 1988–1997. Academic Emergency Medicine. 1999; 6: 1030–1035. DOI: 10.1111/j.1553-2712.1999.tb01188.x 10530662

[2] Fieldston ES, et al. A qualitative assessment of reasons for nonurgent visits to the emergency department: Parent and health professional opinions. Pediatr Emerg Care. 2013; 28(3): 220–225. DOI: 10.1097/PEC.0b013e318248b431 22344210

[3] Grigg A, et al. Factors associated with nonurgent use of pediatric emergency care among Latino families. J Natl Med Assoc. 2013; 105(1): 77–84. DOI: 10.1016/S0027-9684(15)30088-2 23862299

[4] Hoot NR and Aronsky D. Systematic review of emergency department crowding: Causes, effects, and solutions. Annals of Emergency Medicine. 2008; 52: 126–136. DOI: 10.1016/j.annemergmed.2008.03.014 18433933 PMC7340358

[5] Pines JM and Hollander JE. Emergency department crowding is associated with poor care for patients with severe pain. Annals of Emergency Medicine. 2008; 51: 1–5. DOI: 10.1016/j.annemergmed.2007.07.008 17913299

[6] Campos-Miño S. Pediatric intensive care in Latin America. Medicina Intensive. 2012; 36(1): 3–10. DOI: 10.1016/j.medin.2011.07.004 21906846

[7] Baez AA, et al. Knowledge and attitudes of the out-of-hospital emergency care consumers in Santo Domingo, Dominican Republic. 2008 DOI: 10.1017/S1049023X00006038 18935954

[8] Thind A. Diarrhea in the Dominican Republic: Determinants of the utilization of children’s health services. Journal of Tropical Pediatrics. 2003 DOI: 10.1093/tropej/49.2.93 12729291

[9] Thind A and Andersen R. Respiratory illness in the Dominican Republic: What are the predictors for health services utilization of young children? Social Science & Medicine. 2003; 56(6): 1173–1182. DOI: 10.1016/S0277-9536(02)00116-8 12600356

[10] Yoffe SJ. A reduction in emergency department use by children from a parent educational intervention. Family medicine. 2011; 43(2): 106–111. 21305425

[11] Morrison AK, et al. The relationship between parent health literacy and pediatric emergency department utilization: a systematic review. Acad Pediatr. 2013; 13(5): 421–429. DOI: 10.1016/j.acap.2013.03.001 23680294 PMC3808118

[12] Porro F, Monzani V and Folli C. Reasons for inappropriate attendance of the emergency room in a large metropolitan hospital. European Journal of Internal Medicine. 2013; 24: 13–14. DOI: 10.1016/j.ejim.2012.11.016 23265994

[13] Kiechle ES, Hnat AT, Norman KE, Viera AJ, DeWalt DA and Brice JH. Comparison of brief health literacy screens in the emergency department. Journal of Health Communication. 2015; 20(5): 539–545. DOI: 10.1080/10810730.2014.999893 25807061

[14] World Health Organization. Emergency Triage Assessment and Treatment: Manual for Participants 2005 http://apps.who.int/iris/bitstream/10665/43386/1/9241546875_eng.pdf.

[15] Western Cape Government Health. The South African Triage Scale (SATS): Training Manual 2012 http://emssa.org.za/wp-content/uploads/2011/04/SATS-Manual-A5-LR-spreads.pdf.

